# Applicability of Yale-Brown Obsessive-Compulsive Scale Modified for Body Dysmorphic Disorder (BDD-YBOCS) for the Assessment of Patients Eligible for Facial Esthetic Surgery: A Scoping Review

**DOI:** 10.1055/s-0046-1819563

**Published:** 2026-04-30

**Authors:** Arthur Ricardo Carminatti Ortina, Carolina Rodrigues Laranjeira Vilar, Odin Ferreira do Amaral Neto, Rogerio Hamerschmidt, Maria Theresa Costa Ramos Oliveira

**Affiliations:** 1 Departament of Otorhinolaryngology, Setor de Ciências da Saúde, Universidade Federal do Paraná, Curitiba, PR, Brazil; 2 Núcleo de Ensino e Pesquisa (NEP), Hospital Instituto Paranaense de Otorrinolaringologia (IPO), Curitiba, PR, Brazil

**Keywords:** body dysmorphic disorders, plastic surgery, rhinoplasty

## Abstract

**Introduction:**

Although the Yale-Brown Obsessive-Compulsive Scale Modified for Body Dysmorphic Disorder (BDD-YBOCS) is a complete questionnaire for evaluating BDD, there are few studies in the literature to investigate or screen for dysmorphism in patients undergoing facial plastic surgery.

**Objective:**

To map and evaluate facial plastic studies that used this questionnaire to research body dysmorphism in their populations. The secondary objectives were to review the criteria and questions assessed by the questionnaire and their applicability.

**Methods:**

The study selection criteria involved studies that used the BDD-YBOCS in screening studies for dysmorphism in patients who were candidates for or underwent facial plastic surgery. Literature search was carried out in PubMed/electronic database, database Google Scholar data, and Web of Science by the authors. Two independent researchers conducted a citation search of relevant literature. To select appropriate patients for esthetic procedures, validated preoperative BDD screening tools should be used.

**Results:**

Historically, patients with BDD had contraindications for esthetic procedures and surgeries. However, recent evidence supports more refined decision-making based on disorder severity and patients' overall level of functioning.

**Conclusion:**

It is reaffirmed that esthetic surgery is not effective in treating BDD, despite declared patient satisfaction. Therefore, the psychological disorder must be treated first because surgical treatment without prior psychological treatment may result in dangerous consequences for surgeons.

## Introduction

The Yale-Brown Obsessive-Compulsive Scale Modified for Body Dysmorphic Disorder (BDD-YBOCS) is a semistructured, graded, rater-administered questionnaire consisting of 12 items designed to measure the severity of BDD symptoms over a week prior to the test, being considered the gold standard for measuring the severity of this disorder. 1 The measures were initially adapted from the YBOCS, a graded questionnaire mostly used to measure the severity of Obsessive Compulsive Disorder, due to the similarity of symptoms. 2

Since its development, the BDD-YBOCS, administered by an evaluator, has been the main tool used to assess the severity of BDD in research focused on treatment outcomes, including clinical trials that evaluate the efficacy of pharmacological and cognitive-behavioral therapies. 3 4

The BDD-YBOCS consists of 12 items designed to assess symptom severity, ranging from “none” to “very severe.” The first five items evaluate various aspects, including the individual's preoccupation with their appearance (e.g., the amount of time spent focusing on perceived esthetic flaws), the impact on psychosocial functioning, and the distress caused by these perceived defects. Additionally, these items measure the extent to which the person avoids activities related to appearance, as well as the nature of their thoughts and their perceived ability to control these concerns. 4

Items 6 through 10 evaluate behaviors such as repetitive actions and rituals, which are linked to concerns about BDD (e.g., repeatedly checking one's appearance in the mirror), using products for camouflage, excessive grooming, seeking reassurance, and similar behaviors. Item 11 examines the individual's awareness of their condition, specifically how much they acknowledge the problematic and unnecessary nature of their daily symptoms. Item 12 measures the impact of BDD symptoms on their psychosocial well-being. 4

The BDD-YBOCS has shown strong psychometric properties. 1 5 The initial validation study revealed high reliability (including interrater, test-retest, and internal consistency), as well as good convergent and divergent validity, and demonstrated its sensitivity to changes in patients with BDD undergoing treatment. 4 5

The factor analysis identified three primary factors: 1) the Diagnostic and Statistical Manual of Mental Disorders (DSM) criteria for BDD, which involves an intense preoccupation with a physical flaw, whether minor or barely noticeable; 2) compulsive behaviors related to appearance, such as frequent attempts to check, correct, or conceal physical concerns; and 3) attempts to control and resist symptoms associated with appearance, including worry and related behavioral manifestations. 4

The BDD-YBOCS, in particular, offers an empirically established cutoff score (≥ 20) for diagnosis and collects detailed, objective data on symptoms and their progression throughout treatment. 1 6 This allows for a more straightforward comparison of symptom severity and treatment outcomes across individuals. Despite its strong reliability, convergent validity, and clinical utility, using the BDD-YBOCS requires significant time from the evaluator and extensive training for proper implementation. Depending on the complexity of symptoms and the medical professional's experience, interviews typically take between 10 and 40 minutes to complete. 4

Despite being a complete questionnaire for the assessment of BDDs, which can be applied by an evaluator or by the participant themselves, there are few studies in the literature to investigate or screen for dysmorphism in patients undergoing facial plastic surgery. Thus, the present scoping review was conducted in order to map and evaluate facial plastic studies that used the BDD-YBOCS questionnaire to research dysmorphism in their populations. The secondary objective was to review the criteria and questions assessed by the questionnaire and their applicability.

The BDD is a concerning condition that has only recently been systematically studied, with limited data available in the literature. Additionally, there has been an observed increase in the demand for esthetic procedures by patients with dysmorphic profiles, despite some cases still being underdiagnosed. Given that the authors frequently treat this population and use the BDD-YBOCS to assess eligibility for esthetic surgeries, they were prompted to conduct this scoping review.

## Methods

A scope review was planned according to the Preferred Reporting Items for Systematic Reviews and Meta-Analyses extension for Scoping Reviews (PRISMA-ScR) using the structure described by Arksey and O'Malley. 7

A review protocol was developed prior to the study and it was registered on the Open Science Framework (OSF) website. 8

An initial search was performed to help identify appropriate search terms. Keywords such as ' *dysmorphism* , *body dysmorphic disorder* , *plastic surgery* , *facial plastic surgery* , *rhytidoplasty* , *rhinoplasty* , *otoplasty* , *Yale-Brown Obsessive-Compulsive Scale* , and *Yale-Brown Obsessive-Compulsive Scale modified for Body Dysmorphic Disorder (BDD-YBOCS)* , were used along with Medical Subject Headings (MeSH).

The literature search was carried out on PubMed, Google Scholar, and Web of Science databases by the authors. Two independent researchers conducted a citation search of the relevant literature.

**Fig. 1:**
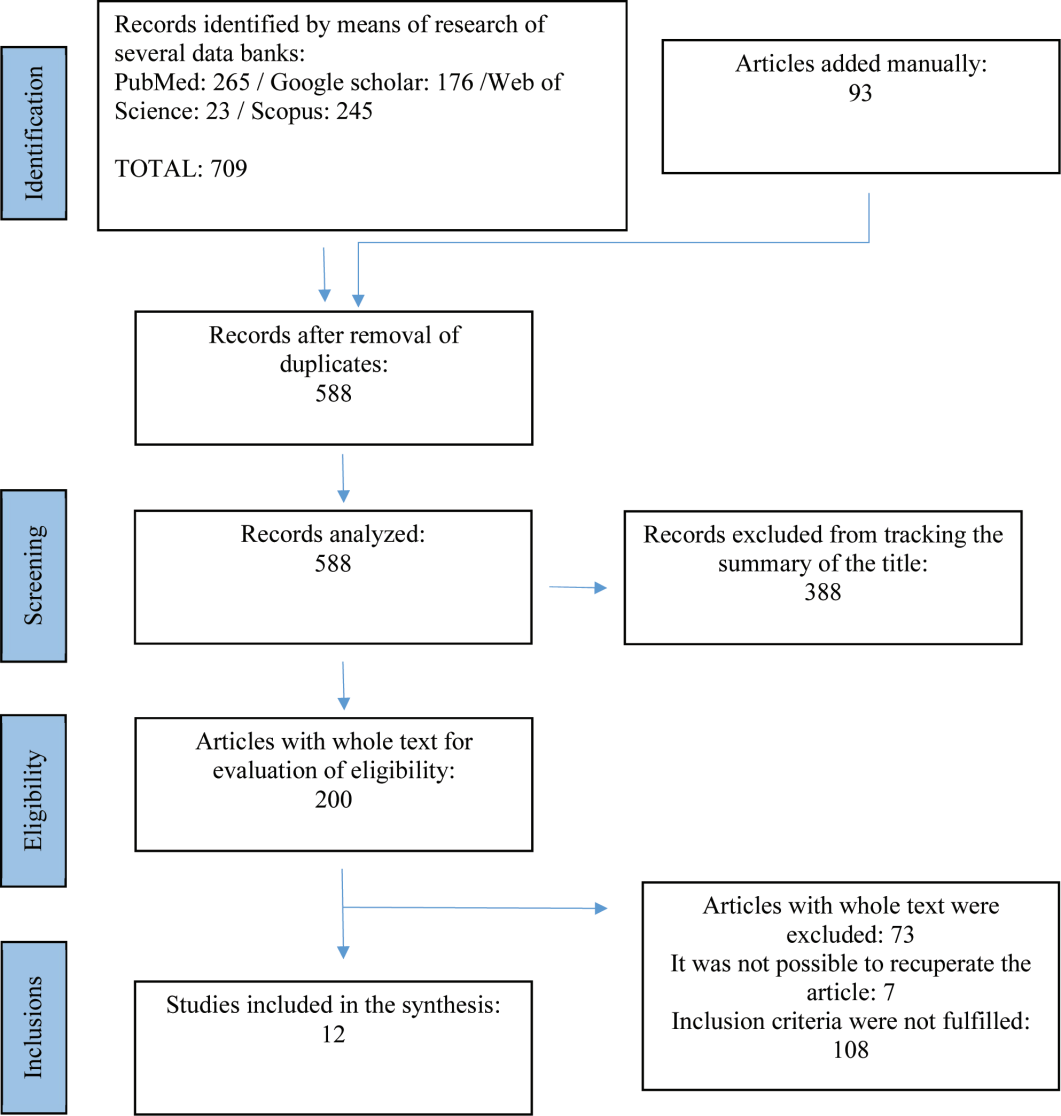
Preferred Reporting Items for Systematic Reviews and Meta-Analyses (PRISMA) flowchart of the article-selection process.

A PRISMA flowchart describing the article selection process and the number of studies produced at each step is provided in Fig. 1 . 9

Studies considered relevant for the review were evaluated as for titles and abstracts. Irrelevant studies were discarded. Selection of abstracts took into account the study inclusion criteria, which involved those that used the Brazilian version of the BDD-YBOCS in screening studies for dysmorphism in patients eligible for facial plastic surgery or who already underwent it. We excluded studies that did not use the BDD-YBOCS or assess facial plastic surgery candidates or patients.

**Fig. 2:**
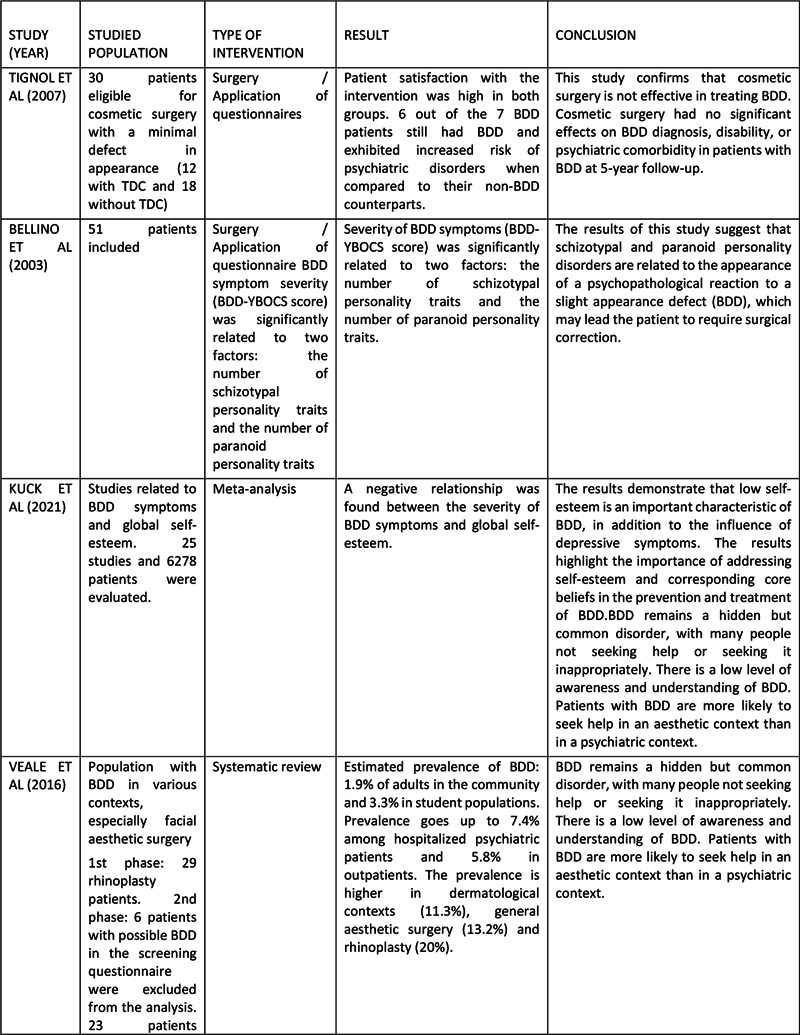
Table of eligible articles.

Articles with chosen abstracts were fully assessed to a full assessment of the entire article. A form was developed to extract relevant information for the research. Two of the evaluators mapped the data independently to decide whether or not each publication should be included in the review flowchart. All studies were referenced. The articles considered eligible were evaluated regarding the type of surgery, type of study, studied population and results. A third reviewer reviewed the eligible articles and made some recommendations. Afterwards, the studies included formed a final group of chosen studies ( Fig. 2 ).

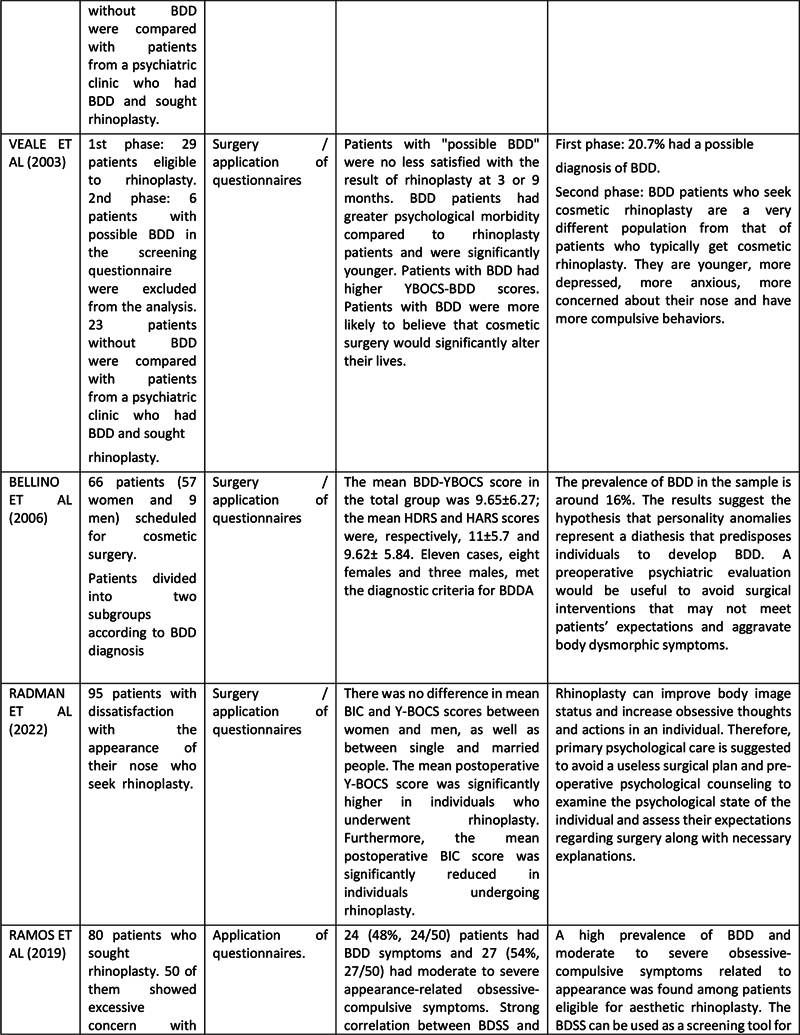

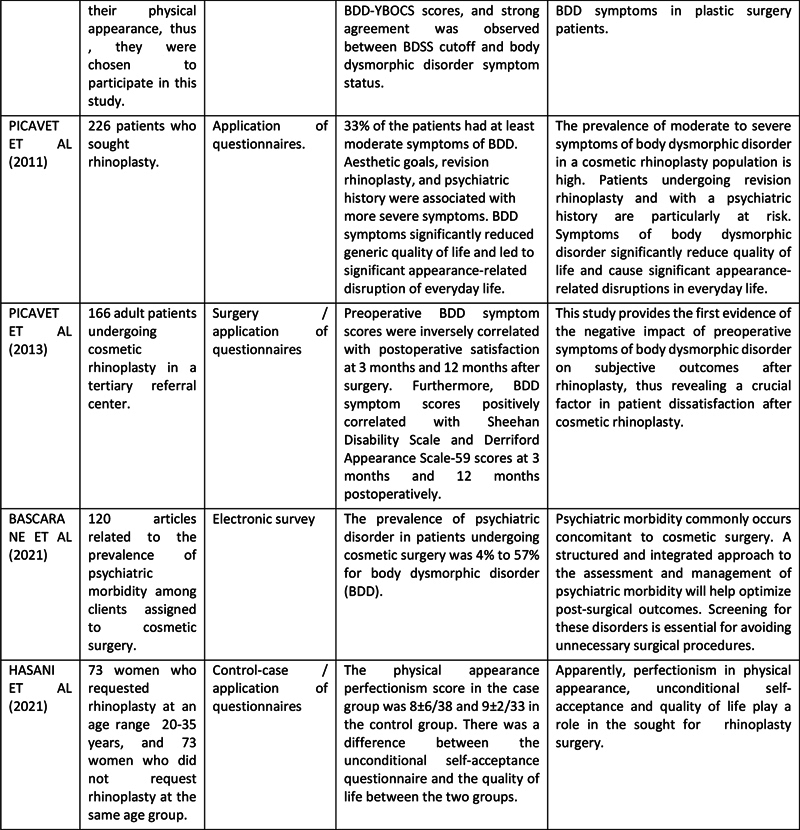





## Results

A prospective study has shown that cosmetic surgery is not an effective treatment for BDD, even though patients often report satisfaction with the results. At a 5-year follow-up, cosmetic surgery did not significantly impact diagnosis, disability, or psychiatric comorbidities in patients with BDD. 10 The reported satisfaction after surgery may explain why some plastic surgeons do not fully adhere to the recommendation against performing esthetic procedures on this cohort. 11

The disorder is also linked to various psychiatric conditions. Bellino et al. 9 found that schizotypal and paranoid personality disorders are associated with the development of a psychopathological response to even a minor physical flaw (BDD), which may lead individuals to seek surgical intervention. Similarly, Kuck et al. 12 highlighted that low self-esteem is a key feature of BDD, along with the influence of depressive symptoms. In fact, negative self-evaluation in this disorder extends beyond physical appearance and affects other aspects of the individual's self-concept. This emphasizes the importance of addressing low self-esteem and underlying core beliefs in both the prevention and treatment of BDD.

The work of Veale et al. 13 highlights that BDD remains a hidden but common disorder, in which many people do not seek help or do it inappropriately. There is still a low level of awareness and understanding among the public, as well as healthcare professionals. Patients with BDD are more likely to seek help in an esthetic setting than in a psychiatric setting. 13

Research by Veale et al. 14 revealed that patients with BDD had greater psychological morbidity when compared to a control group also seeking rhinoplasty, and they were significantly younger. This cohort had higher scores on the BDD-YBOCS, anxiety, and depression scales. Patients with BDD were also more likely to believe that cosmetic surgery would significantly change their lives (for instance, getting a new partner or a job). The BDD patients seeking cosmetic rhinoplasty make up a very different population from other groups who typically undergo this cosmetic surgery. They are significantly younger, more depressed, more anxious, more concerned about their nose, and have more compulsive behaviors. 14

In a study with 66 participants by Bellino et al., 15 the majority (∼70%) had applied for esthetic procedures to correct facial defects. The prevalence of BDD in the sample was around 16%. Thus, the results suggest the hypothesis that personality anomalies represent a diathesis, according to which individuals are predisposed to develop this disorder. If this hypothesis is confirmed by longitudinal investigations, the clinical implications should be considered. A preoperative psychiatric evaluation would be useful to avoid surgical interventions that may not meet patients' expectations and aggravate body dysmorphic symptoms. 15

In line with esthetic rhinoplasty, Ramos et al. found a high prevalence of BDD and moderate to severe obsessive-compulsive symptoms related to appearance among patients eligible the surgery. 16 The same was reported by another study. 17 Furthermore, Radman and Pourhoseinali's study results demonstrated that rhinoplasty can improve the state of body image and increase obsessive thoughts and actions in individuals. Therefore, they suggested primary psychological care to avoid a futile surgical plan and preoperative psychological counseling for assessing the patient's psychological state and their expectations regarding surgery along with necessary explanations. 18

Picavet et al. 19 highlighted that preoperative BDD symptom scores were inversely correlated with satisfaction 3-months postoperatively. This study provides the first evidence of the negative impact of preoperative symptoms of BDD on subjective outcome after rhinoplasty, thus revealing a crucial factor in patient dissatisfaction after cosmetic rhinoplasty. 19

Finally, Bascarane et al. 20 confirm the high prevalence of psychiatric disorder in patients undergoing cosmetic surgery, from 4 to 57% for BDD patients. Screening for these disorders is essential to avoid unnecessary surgical procedures, as well as to ensure time management of psychiatric comorbidity. 20

## Discussion

As a mental disorder, BDD is characterized by preoccupation with a small or imaginary defect in someone's appearance. Alternatively, there may be a minor physical abnormality with comparatively excessive concern. 14

There are frequent comorbidities in BDD, especially for depression, social phobia, and obsessive-compulsive disorder (OCD). Any part of the body may be involved, although concern most commonly focuses on the skin, hair, or facial features (eyes, eyelids, nose, lips or mouth, jaw or chin). The nature of the concern may change over time, and this may explain why, after cosmetic surgery, the patient's focus may shift to another area of the body. 21

Patients with BDD have frequently been seen seeking cosmetic surgical enhancement, with a reported prevalence of 6 to 15%. Studies published on the general population tend to show a prevalence of 0.7 to 2.3%. 11

In recent years, there has been a significant rise in the number of individuals seeking cosmetic procedures. In 1992, over 400,000 Americans underwent cosmetic surgery. By 2015, a total of 21 million cosmetic procedures, both surgical and nonsurgical, were performed globally, with 15.9 million of these taking place in the United States. 22 23 In the United Kingdom (UK), the number of procedures has surged by 300% since 2002. 24 The countries with the highest volume of both surgical and nonsurgical procedures are the United States, Brazil, South Korea, India, and Mexico. 23

In a study by Bouman et al., 25 an online survey was conducted with 173 members from Dutch professional associations in esthetic plastic surgery, dermatology, and esthetic medicine. The results showed that about ⅔ of dermatological surgeons viewed BDD as a contraindication for cosmetic procedures. These practitioners argued that since this disorder is primarily an issue of body image, undergoing cosmetic treatments is unlikely to result in significant or lasting improvement. 25

The psychological disorder must be treated first, as starting with a surgical treatment can result in dangerous or even fatal consequences for the surgeon. 26 27 Dissatisfied patients may attempt to retaliate against the surgeon, who they believe aggravated their defect. 27 This can take the form of legal proceedings, physical assault or, in some cases, homicide. 27 One study reported that 2% of plastic surgeons were physically threatened by patients with BDD and 10% received threats of violence and legal action. 27 28 In another study, 40% of plastic surgeons reported having been threatened by a patient with BDD. 24 Since 1991, there have been reports of three plastic surgeons being murdered by patients with BDD who were dissatisfied with their surgical results. 27

Due to the high prevalence of BDD in patients who are eligible for facial plastic surgery, it is necessary to learn about individual cases and apply questionnaires that can screen patients with the disorder, so they can be referred for prior or adjuvant treatment. The BDD-YBOCS is a specific instrument that screens and measures the severity of BDD symptoms. The Brazilian Portuguese version of this scale, validated in a sample of esthetic surgery patients (n = 63), presents excellent internal consistency. 29

Once preoperative evaluation identifies a potential diagnosis of BDD, a multidisciplinary team should be involved in confirming the diagnosis, considering evidence-based treatments (i.e., cognitive behavioral therapy and selective serotonin reuptake inhibitors), and in determining the suitability of the procedure in question. 30 31

Considerations regarding the appropriateness of the procedure should include a categorization of mild to moderate or severe disease, the patient's medical history, the procedure and defect under consideration, anticipated satisfaction, patient safety, and surgeon comfort. 32

In our medical practice, we have increasingly observed a demand for esthetic procedures, including among this group of patients, most of whom have not initiated treatment, consequently contributing to a rise in the number of surgeries and their potential complications, frustrations, medical errors, and insecurity. The applicability of the BDD-YBOCS for assessing patients eligible for facial esthetic surgery can help prevent such misfortunes and guide patients toward the recommended treatment. Additionally, this review serves as a valuable resource to identify knowledge gaps and guide future reviews.

## Conclusion

To select appropriate patients for esthetic surgeries, validated preoperative BDD screening tools must be used, such as the BDD-YBOCS. It has also been validated for the Portuguese language, in addition to establishing multidisciplinary team relationships, especially with colleagues in the mental health area.

Historically, patients with BDD had contraindications for cosmetic procedures and surgeries. However, recent findings advocate for a more nuanced approach to decision-making, taking into account the condition's severity and patients' overall functional status.

After the diagnosis and treatment of BDD, the procedure under analysis must be reassessed. This includes BDD degree, medical history, expected satisfaction, patient's safety, and surgeon's comfort in carrying it out.

Additional prospective research is necessary to evaluate the outcomes and efficacy of esthetic procedures in individuals with BDD.

## References

[1] Phillips K A Body dysmorphic disorder: common, severe and in need of treatment research Psychother Psychosom 2014 83 06 325 329 10.1159/000366035 25322928

[2] Goodman W K Price L H Rasmussen S A The Yale-Brown Obsessive Compulsive Scale. I. Development, use, and reliability Arch Gen Psychiatry 1989 46 11 1006 1011 10.1001/archpsyc.1989.01810110048007 2684084

[3] Harrison A De la Cruz L F Enander J Radua J Mataix-Cols D Cognitive-behavioral therapy for body dysmorphic disorder: A systematic review and meta-analysis of randomized controlled trials Clin Psychol Rev 2016 48 43 51 10.1016/j.cpr.2016.05.007 27393916

[4] Patel T A Summers B J Wilver N L Cougle J R Reliability and Validity of the Self-Report Version of the Yale-Brown Obsessive-Compulsive Scale Modified for Body Dysmorphic Disorder Assessment 2023 30 06 1935 1946 10.1177/10731911221124341 36114713

[5] Phillips K A Hollander E Rasmussen S A Aronowitz B R DeCaria C Goodman W K A severity rating scale for body dysmorphic disorder: development, reliability, and validity of a modified version of the Yale-Brown Obsessive Compulsive Scale Psychopharmacol Bull 1997 33 01 17 22 9133747

[6] Wilhelm S Phillips K A Didie E Modular cognitive-behavioral therapy for body dysmorphic disorder: a randomized controlled trial Behav Ther 2014 45 03 314 327 10.1016/j.beth.2013.12.007 24680228 PMC4283214

[7] Arksey H O'Malley L Scoping studies: towards a methodological framework Int J Soc Res Methodol 2005 8 01 19 32 10.1080/1364557032000119616

[8] Foster E D Deardorff A Open Science Framework (OSF) J Med Libr Assoc 2017 105 02 203 206 10.5195/jmla.2017.88

[9] Bellino S Zizza M Paradiso E Body dysmorphic disorder and personality disorders: A clinical investigation in patients seeking cosmetic surgeryJ Psychopathol 2003;2 Available from:https://old.jpsychopathol.it/article/disturbo-da-dismorfismo-corporeo-e-disturbi-di-personalita-unindagine-clinica-in-pazienti-della-chirurgia-estetica/10.1016/j.psychres.2005.06.010 16914206

[10] Tignol J Biraben-Gotzamanis L Martin-Guehl C Grabot D Aouizerate B Body dysmorphic disorder and cosmetic surgery: evolution of 24 subjects with a minimal defect in appearance 5 years after their request for cosmetic surgery Eur Psychiatry 2007 22 08 520 524 10.1016/j.eurpsy.2007.05.003 17900876

[11] Pavan C Simonato P Marini M Mazzoleni F Pavan L Vindigni V Psychopathologic aspects of body dysmorphic disorder: a literature review Aesthetic Plast Surg 2008 32 03 473 484 10.1007/s00266-008-9113-2 18224271

[12] Kuck N Cafitz L Bürkner P C Hoppen L Wilhelm S Buhlmann U Body dysmorphic disorder and self-esteem: a meta-analysis BMC Psychiatry 2021 21 01 310 10.1186/s12888-021-03185-3 34130638 PMC8207567

[13] Veale D Gledhill L J Christodoulou P Hodsoll J Body dysmorphic disorder in different settings: A systematic review and estimated weighted prevalence Body Image 2016 18 168 186 10.1016/j.bodyim.2016.07.003 27498379

[14] Veale D Kinderman P Riley S Lambrou C Self-discrepancy in body dysmorphic disorder Br J Clin Psychol 2003 42(Pt 2):157 169 10.1348/014466503321903571 12828805

[15] Bellino S Zizza M Paradiso E Rivarossa A Fulcheri M Bogetto F Dysmorphic concern symptoms and personality disorders: a clinical investigation in patients seeking cosmetic surgery Psychiatry Res 2006 144 01 73 78 10.1016/j.psychres.2005.06.010 16914206

[16] Ramos T D Brito MJAd Suzuki V Y Sabino M Neto Ferreira L M High Prevalence of Body Dysmorphic Disorder and Moderate to Severe Appearance-Related Obsessive-Compulsive Symptoms Among Rhinoplasty Candidates Aesthetic Plast Surg 2019 43 04 1000 1005 10.1007/s00266-018-1300-1 30607575

[17] Picavet V A Prokopakis E P Gabriëls L Jorissen M Hellings P W High prevalence of body dysmorphic disorder symptoms in patients seeking rhinoplasty Plast Reconstr Surg 2011 128 02 509 517 10.1097/PRS.0b013e31821b631f 21788842

[18] Radman M Pourhoseinali L Effect of rhinoplasty on changing body images in candidates for surgery J Family Med Prim Care 2022 11 09 5535 5539 10.4103/jfmpc.jfmpc_2116_21 36505585 PMC9731004

[19] Picavet V A Gabriëls L Grietens J Jorissen M Prokopakis E P Hellings P W Preoperative symptoms of body dysmorphic disorder determine postoperative satisfaction and quality of life in aesthetic rhinoplasty Plast Reconstr Surg 2013 131 04 861 868 10.1097/PRS.0b013e3182818f02 23249985

[20] Bascarane S Kuppili P P Menon V Psychiatric Assessment and Management of Clients Undergoing Cosmetic Surgery: Overview and Need for an Integrated Approach Indian J Plast Surg 2021 54 01 8 19 10.1055/s-0040-1721868 33854274 PMC8034989

[21] Veale D Advances in a cognitive behavioural model of body dysmorphic disorder Body Image 2004 1 01 113 125 10.1016/S1740-1445(03)00009-3 18089145

[22] Lee K Guy A Dale J Wolke D Adolescent Desire for Cosmetic Surgery: Associations with Bullying and Psychological Functioning Plast Reconstr Surg 2017 139 05 1109 1118 10.1097/PRS.0000000000003252 28445361

[23] Valikhani A Goodarzi M A Contingencies of Self-Worth and Psychological Distress in Iranian Patients Seeking Cosmetic Surgery: Integrative Self-Knowledge as Mediator Aesthetic Plast Surg 2017 41 04 955 963 10.1007/s00266-017-0853-8 28374299

[24] Ziglinas P Menger D J Georgalas C The body dysmorphic disorder patient: to perform rhinoplasty or not? Eur Arch Otorhinolaryngol 2014 271 09 2355 2358 10.1007/s00405-013-2792-6 24190759

[25] Bouman T K Mulkens S Van der Lei B Cosmetic Professionals' Awareness of Body Dysmorphic Disorder Plast Reconstr Surg 2017 139 02 336 342 10.1097/PRS.0000000000002962 28121864

[26] Alavi S S Maracy M R Jannatifard F Eslami M The effect of psychiatric symptoms on the internet addiction disorder in Isfahan's University students J Res Med Sci 2011 16 06 793 800 22091309 PMC3214398

[27] Sweis I E Spitz J Barry D R Jr Cohen M A Review of Body Dysmorphic Disorder in Aesthetic Surgery Patients and the Legal Implications Aesthetic Plast Surg 2017 41 04 949 954 10.1007/s00266-017-0819-x 28204935

[28] Wang Q Cao C Guo R Avoiding Psychological Pitfalls in Aesthetic Medical Procedures Aesthetic Plast Surg 2016 40 06 954 961 10.1007/s00266-016-0715-9 27761610

[29] Brito MJAd Nahas F X Cordás T A Tavares H Ferreira L M Body Dysmorphic Disorder in Patients Seeking Abdominoplasty, Rhinoplasty, and Rhytidectomy Plast Reconstr Surg 2016 137 02 462 471 10.1097/01.prs.0000475753.33215.8f 26818280

[30] Bowyer L Krebs G Mataix-Cols D Veale D Monzani B A critical review of cosmetic treatment outcomes in body dysmorphic disorder Body Image 2016 19 1 8 10.1016/j.bodyim.2016.07.001 27517118

[31] Brito MJAd Felix GAAd Nahas F X Body dysmorphic disorder should not be considered an exclusion criterion for cosmetic surgery J Plast Reconstr Aesthet Surg 2015 68 02 270 272 10.1016/j.bjps.2014.09.046 25456281

[32] Tadisina K K Chopra K Singh D P Body dysmorphic disorder in plastic surgery Eplasty 2013 13 ic48 23814639 PMC3693597

